# The Effects of RBP4 and Vitamin D on the Proliferation and Migration of Vascular Smooth Muscle Cells via the JAK2/STAT3 Signaling Pathway

**DOI:** 10.1155/2022/3046777

**Published:** 2022-01-17

**Authors:** Wan Zhou, Wei Wang, Xiao-Jing Yuan, Chun-Chun Xiao, Yan Xing, Shan-Dong Ye, Qiang Liu

**Affiliations:** ^1^Laboratory for Diabetes, Department of Endocrinology, The First Affiliated Hospital of USTC, Division of Life Sciences and Medicine, University of Science and Technology of China, Hefei, 230001 Anhui Province, China; ^2^The First Affiliated Hospital of USTC, Division of Life Sciences and Medicine, University of Science and Technology of China, Hefei 230001, China; ^3^Institute on Aging and Brain Disorders, The First Affiliated Hospital of USTC, Division of Life Sciences and Medicine, Hefei National Laboratory for Physical Sciences at the Microscale, University of Science and Technology of China, Hefei 230026, China

## Abstract

Abnormal proliferation and migration of vascular smooth muscle cells (VSMCs) are one of the main causes of the development of diabetic atherosclerotic process. The aim of our study was to assess the role of RBP4 in the proliferation and migration of VSMCs and the inhibitory effect of vitamin D on the mechanisms. In an in vivo experiment, rats were randomly classified into 6 groups: the control group, diabetic rats, diabetic atherosclerotic rats (diabetic rats intraperitoneally injected with RBP4), diabetic atherosclerotic rats treated with 0.075 *μ*g kg^−1^ d^−1^ vitamin D, 0.15 *μ*g kg^−1^ d^−1^ vitamin D and 0.3 *μ*g kg^−1^ d^−1^ vitamin D. We found that the levels of JAK2, STAT3, cylinD1, and Bcl-2 were increased in diabetic atherosclerotic rats, and these increases were improved after vitamin D supplementation. Furthermore, to investigate the underlying molecular mechanisms, cells were cultured with glucose in the presence of RBP4 and the absence of RBP4, respectively, and vitamin D of different concentrations and different intervention times was simultaneously adopted. The proliferation and migration of VSMCs was enhanced and the levels of JAK2, STAT3, cyclinD1, and Bcl-2 were increased in the cells transfected with RBP4 overexpression plasmid. Moreover, vitamin D supplementation was detected to lower the expressions of JAK2, STAT3, cyclinD1, and Bcl-2 and inhibit the abnormal proliferation of VSMCs caused by the RBP4/JAK2/STAT3 signaling pathway. RBP4 can promote the proliferation and migration of VSMCs and contributes to the development of diabetic macroangiopathy via regulating the JAK2/STAT3 signaling pathway. This mechanism of RBP4 can be inhibited by vitamin D supplementation.

## 1. Introduction

Diabetic macroangiopathy, a specific form of atherosclerosis secondary to diabetes, causes cerebro-cardiovascular diseases, which are correlated with high mortality and morbidity in diabetic patients. Previous studies have shown that subclinical inflammation and insulin resistance play significant roles in the development of atherosclerosis and diabetes mellitus (DM) [1, 2]. In addition, the role of adipocytokines in the pathological physiology of diabetic macroangiopathy has attracted considerable attention of the scientific community in recent years. It is known that obesity-activated adipocytes release adipocytokines such as leptin and adiponectin, which can induce energy balance, metabolic regulation, and immunoregulation. Retinol binding protein 4 (RBP4), an adipokine derived from adipocyte and hepatocyte, is responsible for transporting retinol to systemic tissues [3]. Elevated circulating RBP4 levels were found to contribute to systemic insulin resistance and type 2 diabetes mellitus (T2DM) by inhibiting phosphatidylinositol 3 kinase (PI3K) activity in the skeletal muscles and promoting phosphoenolpyruvate carboxylase (PEPCK) expression in the liver of mouse models, which was first reported by Yang in 2005 [4]. Furthermore, the injection of recombinant RBP4 has been discovered to lead to insulin resistance, whereas genetic deletion of RBP4 enhanced insulin sensitivity [4]. Recent reports have revealed that circulating RBP4 concentrations were high in patients with documented carotid atherosclerosis and were paralleled with the degree of carotid stenosis [5, 6]. In addition, elevated serum levels of RBP4 were associated with the prognosis and severity of acute ischemic stroke. Thus, RBP4 might potentially be used as a diagnostic biomarker of ischemic stroke [7]. RBP4 levels are closely correlated to hypertension in diabetic patients and may be engaged in regulating left ventricular diastolic function in individuals with essential hypertension [8]. In contrast, Mansouri et al. showed that serum levels of RBP4 were not the potential predictors of carotid intima-media thickness (CIMT) in T2DM patients [9]. Moreover, elevated concentrations of RBP4 did not relate to an increased risk of ischemic stroke, which was reported by Rist et al. [10]. Several studies even suggested that increased levels of RBP4 might potentially serve a cardiovascular protective role [11, 12]. Therefore, the precise role of RBP4 was yet unclear, and the association between RBP4 and diabetic macroangiopathy remains controversial.

The imbalance of the proliferation and migration of vascular smooth muscle cells (VSMCs) is a vital mechanism potentially causing the pathological of cardiovascular diseases [13]. The Janus kinase/signal transducers and activators of transcription (JAK/STAT) pathway is an evolutionary signaling cascade that regulates cell proliferation, differentiation, apoptosis, and survival. Signal transducer and activator of transcription 3 (STAT3) is a potential cytoplasmic transcriptional factor that is triggered by phosphorylation. Once phosphorylated by Janus-activated kinases 2 (JAK2), the activated STAT3 dimerizes and translocates to the nucleus, which binds to specific enhancer sequences and ultimately results in gene transcription [14]. Various studies have shown that the activation of JAK2/STAT3 signaling pathway promotes the proliferation of VSMCs and involves in the progression of atherosclerosis by modulating apoptotic regulatory proteins such as B cell lymphoma-2 (Bcl-2) and cyclinD1 [15, 16]. However, the regulatory role of JAK2/STAT3 signaling pathway in the proliferation of the VSMCs has not been previously elucidated under diabetic conditions.

As a steroid hormone, vitamin D is not only a key component in mineral homeostasis and bone metabolism but also plays an essential role in immune modulation and glycometabolism [17]. It is hydroxylated to 25-hydroxyvitamin D (25(OH)D), the stable metabolite for assessing the individual status of vitamin D, first in the liver. Then, 25(OH)D is further converted into 1,25-dihydroxyvitamin D (1,25(OH)_2_D) by renal 1-*α*-hydroxylase. Since the overall prevalence of vitamin D deficiency exceeds 50% worldwide, further investigations of the role of vitamin D in metabolic syndromes and other diseases are warranted [18]. Multiple studies have suggested that lower 25(OH)D levels are correlated with higher risks of cardiovascular diseases [19, 20]. However, it is noteworthy to mention that epidemiological studies focusing on the impact of vitamin D supplementation on diabetic macroangiopathy have produced inconsistent results. For example, a randomized double-blind study indicated that blood glucose levels could be improved in patients with diabetes and coronary heart diseases by prolonging sun exposure and increasing vitamin D supplementation [21]. On the other hand, some randomized controlled trials (RCTs) concluded that vitamin D was ineffective in improving cardiovascular diseases [22]. Thus, in this study, we hoped to gain insight into the possible role of vitamin D in the development of diabetic macroangiopathy.

Our previous findings have indicated that elevated RBP4 levels are involved in the pathogenesis of diabetic atherosclerotic rats via the JAK2/STAT3 signaling pathway and vitamin D can negatively regulate RBP4 [23]. Based on these findings, we speculated that RBP4 could potentially contribute to the development of diabetic atherosclerosis by regulating the proliferation and migration of VSMCs, and vitamin D supplementation has cardioprotective benefits. This study is aimed at determining (1) the role of the JAK2/STAT3 signaling pathway on proliferation and migration of VSMCs in the pathogenesis of diabetic atherosclerosis, (2) the potential cellular mechanism of RBP4 involved in the development of diabetic macroangiopathy, and (3) the impact of vitamin D on the regulation of RBP4 and the proliferation and migration of VSMCs.

## 2. Methods

### 2.1. Animals

**Figure 1:**
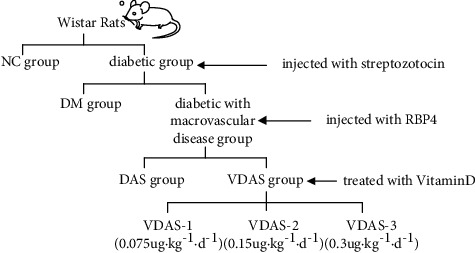
Animal grouping.

The present study was sanctioned by the Ethics Committee of Anhui Provincial Hospital, Medical Institution Animal Care and Research Advisory Committee. According to the Animal Research: Reporting In Vivo Experiments (ARRIVE) Guidelines, the animals were kept under specific pathogen-free (SPF) conditions. A total of 130 male Wistar rats (7-week-old, 190-210 g) were acquired from the Experimental Animal Center of Anhui Medical University (certificate no. SCXK 2017-001) and were randomly classified into the control group fed with normal pellet diet (NC group, *n* = 20) and the diabetic group fed with a high-fat and high-sugar diet (*n* = 110), which was composed of 0.2% propylthiouracil, 10% lard, 3% cholesterol, 0.5% sodium cholate, 5% sugar, and 81.3% chow diet [24]. The rats in the diabetic group were further divided into diabetes without macrovascular disease group (DM group, *n* = 20) and the ones with macrovascular disease group (*n* = 90). After 1 week of acclimatisation, all the rats in the diabetic group were intraperitoneally injected with streptozotocin (STZ) at 30 mg/kg for 5 days, whereas the rats in the NC group were intraperitoneally injected with citric acid-sodium citrate buffer. Additionally, rats in diabetes with macrovascular disease group were intraperitoneally injected with recombinant RBP4 (ab109146, abcam, UK) at 3 *μ*g/g every 12 h for 3 weeks. Glucose tolerance tests were carried out in rats which were fasted for 12 h to confirm T2DM. As a result, 107 rats in the diabetic group were considered T2DM rats with fasting plasma glucose (FPG) over 11.1 mmol/l [25], and 3 rats were died. After 8 weeks of feeding, the rats in the diabetes with macrovascular disease group were randomly divided into 2 subgroups, namely, the diabetic atherosclerosis group treated with vitamin D (VDAS, *n* = 62) and the untreated diabetic atherosclerosis group (DAS, *n* = 25). Rats in the VDAS group were treated with varying levels of vitamin D (0.075 *μ*g kg^−1^ d^−1^ (VDAS-1, *n* = 21), 0.15 *μ*g kg^−1^ d^−1^ (VDAS-2, *n* = 20), and 0.3 *μ*g kg^−1^ d^−1^ (VDAS-3, *n* = 21) dissolved in peanut oil for 8 weeks, respectively. The remaining rats received an equal volume of distilled water daily (Figure 1).

### 2.2. Biochemical Analysis In Vivo

Once the treatment ended, rats were fasted overnight and anesthetized by intraperitoneal injection with sodium pentobarbital (30 mg/kg). Blood was collected from the tail vein of rats. Total cholesterol (TC), low-density lipoprotein-cholesterol (LDL-c), triglyceride (TG), high-density lipoprotein-cholesterol (HDL-c) levels were quantified using an automatic biochemical analyzer (Hitachi 7600-020, Santa Clara, CA, USA). Serum levels of RBP4 and (25(OH)D) were detected by enzyme-linked immunosorbent assay (ELISA) kits (BIOHJSW, USA). Hemoglobin A1c (HbA1C) was measured by high-pressure liquid chromatography (BIO-RAB-D10, USA). Fasting insulin (FINS) were tested by the insulin radioimmunoassay kit (Atom Hi-Tech, China). The insulin sensitivity index (ISI) was calculated according to the formula ISI = Ln1/(FPG × FINS) and the homeostasis model assessment of insulin resistance (HOMA-IR) was estimated by the formula HOMA − IR = FINS × FPG/22.5. The thoracic aortas from the aortic arch were separated and extracted for hematoxylin and eosin (HE) staining, immunofluorescence tests, real-time quantitative reverse transcription polymerase chain reaction (qRT-PCR) analysis, and western blot. After HE staining, 16 slices were selected randomly from each group, and the media thickness (MT) and the lumen diameter (LD) of the tube wall were detected by Image-Pro Plus 6.0 software. Ultimately, the results were averaged together.

### 2.3. Cell Culture and Biochemical Analysis In Vitro

**Figure 2:**
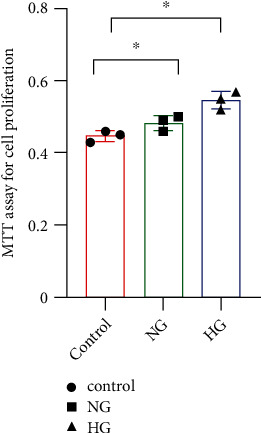
The proliferation of VSMCs by MTT. The ability of cells proliferation were analyzed by MTT (^∗^*P* < 0.05, for HG and NG group compared to the control group).

**Figure 3:**
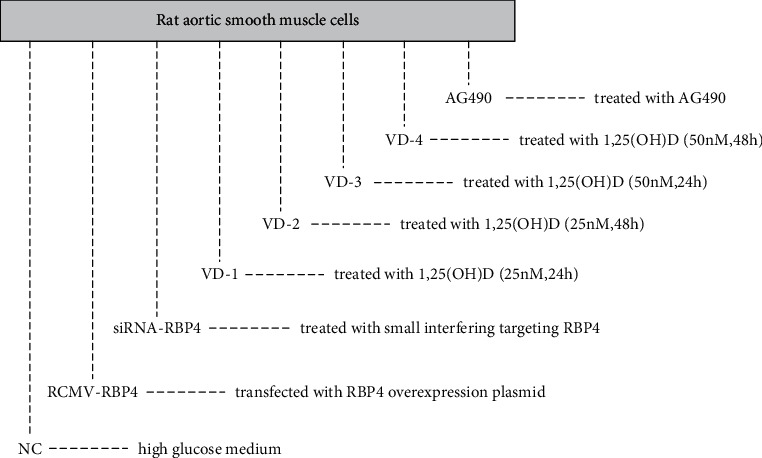
Cell culture in each group.

The rat aortic smooth muscle cells were acquired from the American Type Culture Collection (Manassas, VA, USA). The cells were cultured under standard conditions in DMEM (supplemented with 100 U/ml penicillin, 100 *μ*g/ml streptomycin, 10% fetal bovine serum, and 3.7 g/l NaHCO_3_) without glucose (control), DMEM with normal glucose (NG, 5.5 mM), and DMEM with high glucose (HG, 30 mM), respectively, to choose an appropriate condition by exploring the ability of VSMC proliferation. The proliferation ability of the cells was elevated under 30 mM glucose conditions which we choose for further experiments (Figure 2). VSMCs were then divided into 8 groups (Figure 3): (1) Group NC: the cells kept in high-glucose medium were treated as a control group. (2) Group RCMV-RBP4: the cells were transfected with RBP4 overexpression plasmid (RCMV-RBP4) using Lipofectamine 2000 (Invitrogen, Thermo Fisher Scientific, USA) according to standard procedures and the cells treated with empty plasmid were considered as a control group (RCMV-control). The efficiency of the RBP4 gene overexpression was evaluated by western blot. (3) Group siRNA-RBP4: the cells were treated with small interfering RNA (siRNA) targeting RBP4 (Santa Cruz Biotechnology, Dallas, TX, USA) (siRNA-RBP4). Control siRNA was purchased from Bioneer and used as a negative control (siRNA-control). The interference sequence of RBP4 was evaluated by western blot. (4) Group VD-1: after the cells were transfected with RCMV-RBP4 for 48 h, they were treated with 1,25(OH)_2_D (25 nM) for 24 h. (5) Group VD-2: After transfected with RCMV-RBP4, the cells were treated with 1,25(OH)_2_D (25 nM) for 48 h. (6) Group VD-3: after transfection with RCMV-RBP4, the cells were treated with 1,25(OH)_2_D (50 nM) for 24 h. (7) Group VD-4: after transfection with RCMV-RBP4, the cells were treated with 1,25(OH)_2_D (50 nM) for 48 h. (8): Group AG490: after transfected with RCMV-RBP4, the cells were treated with AG490 (inhibitor of JAK2, 5 × 105 *m*) for 1 h. Cell cycle and apoptosis were assessed with flow cytometric analysis, Transwell assay, scratch test, and MTT assay. The levels of JAK2, STAT3, cyclinD1, and Bcl-2 were analyzed using western blot, qRT-PCR, and immunofluorescence assays.

### 2.4. Western Blot

Protein from tissues and cells was lysed by ice-cold RIPA lysis buffer, and the protein level was measured by the BCA Protein Assay Kit. After centrifugation at 12000 g at 4°C for 5 min, the same quantity of protein was extracted with 10% sodium dodecyl sulfate-polyacrylamide gel electrophoresis (SDS-PAGE) gels and then transferred to polyvinylidene fluoride (PVDF) membranes. Then, the membranes were blocked in 5% skim milk. Afterwards, incubation was carried out with primary antibodies (1 : 1000, anti-RBP4 (Bioss, China); anti- Bcl-2 (Bioss, China) 1 : 300; anti-JAK2 (Bioss, China); 1 : 5000; anti-STAT3 (Bioss, China); 1 : 10000; anti-p-JAK2 (abcam, USA); 1 : 1000; anti-p-STAT3 (abcam, USA); 1 : 300, anti-Cyclin D1 (Bioss, China); 1 : 1000) at 4°C overnight. After washing with TBST for four times, the membranes were then cultured with secondary antibody horseradish peroxidase-conjugated goat anti-rabbit IgG (1 : 10000, Zs-BIO, China) for 1 h. Finally, the protein were visualized with an enhanced chemiluminescence (ECL) detection kit (Thermo, USA), and band intensity was quantified by densitometry with ImageJ software version 1.40 (National Institutes of Health, Bethesda, USA).

### 2.5. Real-Time Quantitative RT-PCR

Total RNA was extracted from tissues and cells by TRIzol reagent (Invitrogen, USA) and reverse-transcribed into cDNA by the PrimeScript™ RT reagent Kit (Takara, Tokyo, Japan). Real-time quantitative RT-PCR amplification was run on the LightCycler 96 Real-Time PCR System (Thermo, USA) in which the Novostart SYBR qPCR SuperMix Plus (Novoprotein, China) was used as the readout. Results were analyzed using the 2^-△△^ cycle threshold (CT) approach to evaluate relative gene expression. The primer sequences are exhibited in Table 1.

### 2.6. ELISA Assay

The levels of serum RBP4 and 25(OH)D were assessed using ELISA Kit (MSKBIO, China) following the instructions given by the manufacturer. The absorbance value (optical density) was detected at 450 nm under a microplate reader (Thermo Fisher Scientific).

### 2.7. Immunofluorescence

Formaldehyde-fixed aortic tissue was incubated in PBS overnight at 4°C. Then, it was embedded in an OCT compound (Tissue-Tek) and serially sectioned on a cryostat. Serial 8 *μ*M sections were stained with HE staining and immunofluorescence. For immunofluorescence, paraffin-embedded sections were deparaffinised with xylene and rehydrated through graded ethanol and then incubated in 10 mmol/l citrate buffer for antigen retrieval. Primary antibodies (1 : 200, anti-JAK2 (abcam, USA); 1 : 100, anti-STAT3 (Bioworld, China); 1 : 200) were used and then incubation of samples were treated with anti-rat Alexa Fluor 488 secondary antibody (abcam, USA) for 30 min at 37°C. Afterwards, the samples were incubated with DAPI solution for 5 min in the dark; they were washed 3 times with PBS and examined under a fluorescence microscope. Then, cells were plated on autoclaved glass coverslips placed in sterile 6-well plates, fixed in 4% paraformaldehyde, permeabilised in 0.1% Triton, and prevented with 10% goat serum. Subsequently, cells were treated with primary antibodies (1 : 200, anti-JAK2 (abcam, USA); 1 : 100, anti-STAT3 (Bioworld, China)) for 1 h at 37°C. After rinsing with PBS 3 times, the fluorescent secondary antibodies were added and the cells were stained as detailed above.

### 2.8. Transwell Migration Assay

A hundred microliters of cell suspension containing 5 × 10^4^ cells of serum-free DMEM were planted into the top chamber of each Transwell insert, whereas the bottom chamber was filled with 500 *μ*l media containing 10% FBS. The top chamber cells were attached 4% paraformaldehyde and stained with a 10-fold-diluted Giemsa solution at room temperature after incubation at 37°C for 48 hours. Later, five randomly visual fields were selected randomly, and the migrated cells were calculated and photographed with a microscope (Olympus, Tokyo, Japan). The relative migration rate was calculated as the fold change of the number of migrated VSMCs when compared with the control group.

### 2.9. MTT Assay

Cell proliferation assay was performed using MTT assay. The VSMCs were harvested into flat-bottomed 96-well plates at a density of 5 × 10^4^/ml. After that, the culture medium was removed and substituted with serum-free DMEM. The cells were then rinsed twice with PBS, followed by incubation along with 10 *μ*l of MTT (Sigma, USA) at 37°C for 4 h in the dark. Consequently, to dissolve MTT crystals, 150 ml of DMSO (Sigma, USA) was added. The absorbance was calculated using an enzyme-labelled instrument (Thermo Fisher Scientific) at 490 nm.

### 2.10. Scratch Assay

VSMCs were cultured until >90% confluent in 6 well dishes and then were incubated for 0 h, 6 h, 24 h, and 48 h to form a cell monolayer in the presence of different culture media. At the incubation times of 0 h, 6 h, 24 h, and 48 h incubations, the scratch across cell monolayer was made with a sterile 10 *μ*l pipette tip by drawing a line through cells perpendicular to the line above. Finally, the wound gap was photographed to measure the scratch width and calculate the relative ratio of width reduction.

### 2.11. Flow Cytometric Analysis

VSMCs in the logarithmic growth phase were collected with EDTA-free trypsin and rinsed two times with 2 ml ice-cold 70% ethanol overnight. After centrifugation, the cells were resuspended in 500 *μ*l PBS containing 10 *μ*g/ml RNase and 50 *μ*g/ml propidium iodide (PI) for 30 minutes at 4°C in the dark. After the cells were rinsed twice with PBS, the cell cycle was detected by NAVIOS cytometer and was analyzed using Kaluza software (Beckman Coulter, USA).

### 2.12. Statistical Analysis

All statistical analyses were carried out using SPSS 23.0 statistical software (IBM, Armonk, USA). Data are expressed as mean ± or median. Student's *t*-test was employed for the comparison of two groups, and one-way analysis of variance (ANOVA) was carried out for the comparison of multiple groups. In addition, Pearson's correlation coefficient was performed to evaluate the relationship between RBP4 and other markers.

## 3. Results

### 3.1. Biochemical Characters of Rats in Each Group

As shown in Table 2, the weights of rats in groups DM and DAS increased at the end of the 16th week compared to those in group NC. The body weights were significantly reduced by the vitamin D supplementation in the VDAS group. The rats in group DAS showed higher levels of LD and MT than the ones in groups NC and DM. Furthermore, vitamin D could decrease the thickness of the carotid artery in a dose-dependent manner. Compared with the NC group and DM groups, LDL-c, TG, TC, HbA1C, HOMA-IR, and RBP4 in DAS group were pronouncedly increased while the levels of 25(OH)D, HDL-c and ISI were decreased (*P* < 0.05). Furthermore, intervention with vitamin D significantly decreased the levels of LDL-c, TG, TC, HOMA-IR, HbA1C, and RBP4 and promoted the levels of 25(OH)D, HDL-c, and ISI in the VDAS group (*P* < 0.05). Thus, vitamin D could significantly reverse the expressions of RBP4 and biochemical parameters in a concentration-dependent manner.

### 3.2. HE Staining of Thoracic Aortas

**Figure 4:**
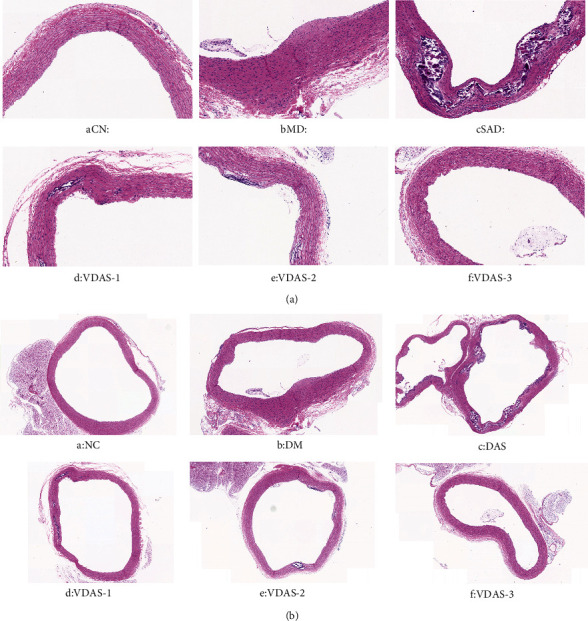
Representative HE staining of thoracic aortas. (a) 10x; scale bar, 200 *μ*m. (b) 20x; scale bar, 50 *μ*m. (A) There were no obvious changes in the structure of aortas in the group NC. (B) The intima became thicker and the VSMCs arranged disorderly in group DM. (C) A large number of VSMCs migrated and proliferated and distinct plaques with abundant lipids and calcification formed in the aortas of the rats in group DAS. (D, E) There were small formations of plagues and calcification on the walls of aortas in the groups VDAS-1 and VDAS-2. (F) The intima was smooth and VSMCs arranged neatly in the group VDAS-3.

As illustrated in Figures 4(a, A–F) and 4(b, A–F), there were no obvious changes in the structure of aortas in the group NC; the intima became thicker and the VSMCs arranged disorderly in group DM; a large amount of VSMCs migrated and proliferated in group DAS and simultaneously distinct plaques with abundant lipids and calcification formed in the aortas of the rats; there were small formations of plagues and calcification on the walls of aortas in the groups VDAS-1 and VDAS-2; the intima of the aorta was smooth and VSMCs arranged neatly in the group VDAS-3. These findings indicated that vitamin D could potentially have a protective effect during the progression of atherosclerosis.

### 3.3. The Expressions of JAK2, STAT3, cylinD1, and Bcl-2 Were Increased in Diabetic Rats Intraperitoneally Injected with RBP4, Which Can Be Improved after Vitamin D Supplementation

**Figure 5:**
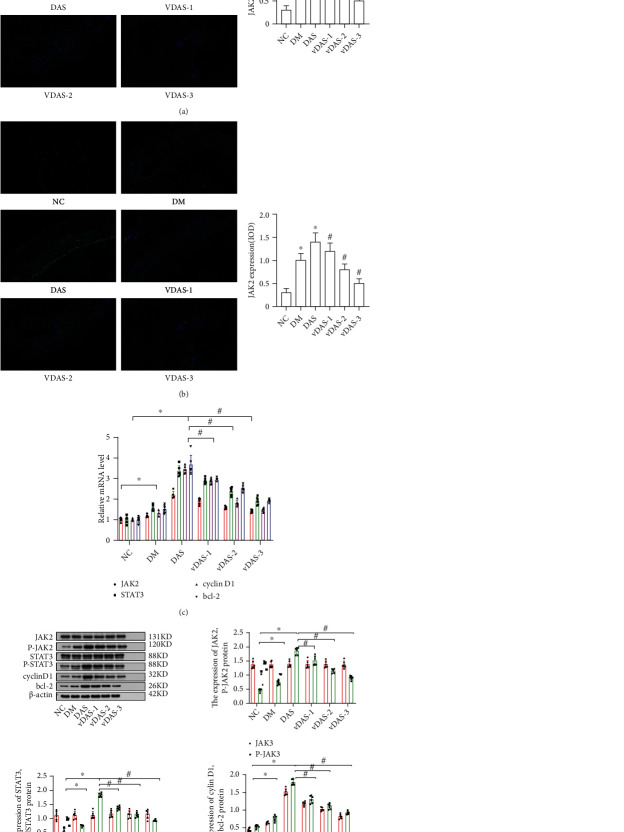
The expressions of JAK2, STAT3, cylinD1 and Bcl-2 were increased in diabetic rats intraperitoneally injected with RBP4, which can be improved after vitamin D supplementation. (a) Immunofluorescence was performed to detect JAK2 (green) in aortic tissue, and blue indicates DAPI-stained nuclei (scale bar, 25 *μ*m). The corresponding OD values are presented (^∗^*P* < 0.05 for group DM and group DAS compared to group NC, ^#^*P* < 0.05 for groups VDAS compared to group DAS). (b) Immunofluorescence was performed to detect STAT3 (green) in aortic tissue, and blue indicates DAPI-stained nuclei (scale bar, 25 *μ*m). The corresponding OD values are presented (^∗^*P* < 0.05 for group DM and group DAS compared to group NC, ^#^*P* < 0.05 for groups VDAS compared to group DAS). (c) The expressions of JAK2, STAT3, cyclinD1, and Bcl-2 were analyzed by qRT-PCR (^∗^*P* < 0.05 for group DM and group DAS compared to group NC, ^#^*P* < 0.05 for groups VDAS compared to group DAS). (d) The expressions of JAK2, STAT3, cyclinD1, and Bcl-2 were analyzed by western blot (^∗^*P* < 0.05 for group DM and group DAS compared to group NC, ^#^*P* < 0.05 for groups VDAS compared to group DAS).

To determine the potential role of RBP4 and vitamin D in the progression of diabetic atherosclerosis, we assessed the expressions of JAK2, STAT3, cylinD1, and Bcl-2 in aortic tissues using different experimental methods. According to the results of immunofluorescence analyses, we found the expressions of JAK2 and STAT3 were markedly increased in the neointima of aortas of rats intraperitoneally injected with RBP4. On the other hand, the expressions were decreased in the neointima of aortas of vitamin D-treated rats (Figures 5(a) and 5(b)). This finding was further confirmed by western blot and qRT-PCR. Quantitative RT-PCR showed that the expressions of JAK2 mRNA in aortic tissues were 1.0 ± 0.08, 1.23 ± 0.12, 3.09 ± 0.15, 2.65 ± 0.13, 1.75 ± 0.13, and 1.55 ± 0.11 in groups NC, DM, DAS, VDAS-1, VDAS-2, and VDAS-3, respectively. Interestingly, the expression of STAT3 mRNA in aortic tissues was higher in the DAS group than those in the NC group and DM group. When compared with group DAS, the mRNA expressions of STAT3 in groups VDAS-1, VDAS-2, and VDAS-3 were significantly lower, and this difference of STAT3 expression was more significant in group VDAS-3. In addition, the mRNA expressions of cyclinD1 and Bcl-2 in the DAS group were significantly increased compared to the NC group and the DM group. After the 8-week treatment with vitamin D, the expressions of cyclinD1 and Bcl-2 decreased in a dose-dependent manner in group VDAS as compared with those in group DAS (Figure 5(c)). Consistently with mRNA levels, western blot (Figure 5(d)) revealed that the protein expressions of p-JAK2, p-STAT3, cylinD1, and Bcl-2 in group DAS were also aberrantly elevated in contrast to control subjects, group DM, and group VDAS. However, no significant difference was observed in JAK2 and STAT3 expressions among the groups. Furthermore, vitamin D exhibited dose-dependent effects in decreasing the protein expressions of p-JAK2, p-STAT3, cylinD1, and Bcl-2. These findings reveal that the expressions of JAK2, STAT3, cylinD1, and Bcl-2 were increased significantly after the diabetic rats were intraperitoneally injected with RBP4, and vitamin D can decrease the expressions in a dose-dependent manner. Pearson's correlation analysis was employed to further validate the relationship between RBP4 and the development of diabetic atherosclerosis. The results indicated that serum RBP4 was positively associated with the LDL-c, TG, HbA1C, TC, FINS, Bcl-2, p-JAK2, cyclinD1, p-STAT3, and HOMA-IR but negatively correlated with 25(OH)D and HDL-c (Table 3).

### 3.4. RBP4 Promotes VSMC Proliferation and Migration under High Glucose Condition, Which Can Be Inhibited by Vitamin D

**Figure 6:**
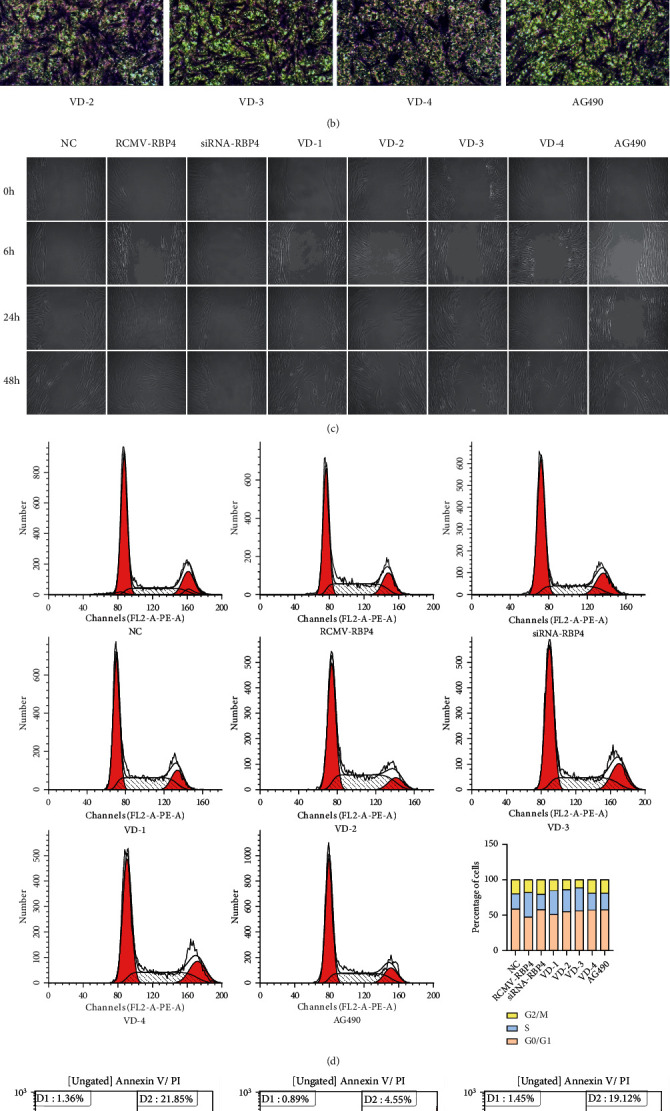
RBP4 can promote VSMCs proliferation and migration under high glucose condition, which can be inhibited by vitamin D. (a) The proliferation ability of VSMCs was measured by MTT, scratch wound-healing, and Transwell migration assay (^∗^*P* < 0.05 for the RCMV-RBP4 group compared to the NC group, ^#^*P* < 0.05 for siRNA-RBP4-, AG490-, and vitamin D-treated groups compared to RCMV-RBP4). (b) VSMC migration ability was measured by Transwell assays. (c) VSMC migration ability was measured by scratch wound assays. (d) Flow cytometry was applied to detect the cell cycle of VSMCs. (e) Flow cytometry was applied to detect the apoptosis rate of VSMCs (^∗^*P* < 0.05 for the RCMV-RBP4 group compared to the NC group, ^#^*P* < 0.05 for siRNA-RBP4-, AG490-, and vitamin D-treated groups compared to RCMV-RBP4).

To elucidate the molecular mechanism of RBP4 and vitamin D in regulating VSMC proliferation, we evaluated the ability of proliferation and migration of VSMCs under high glucose condition. An MTT proliferation assay demonstrated that RBP4 treatment significantly enhanced the proliferation of VSMCs compared to the remaining groups (Figure 6(a)). The Transwell migration assay showed that the number of invaded cells exposed to RCMV-RBP4 was significantly higher than those of cells exposed to siRNA-RBP4. It also revealed that vitamin D could attenuate the proliferation of VSMCs in a time- and dose-dependent manner (Figures 6(a) and 6(b)). Since AG490 is a known inhibitor of JAK2, group AG490 was set up as a reference to clarify the mechanisms by which RBP4 triggers VSMC proliferation. As shown in Figure 6(a), the number of invaded cells exposed to siRNA-RBP4 and AG490 simultaneously was lower than the one of VSMCs exposed to RCMV-RBP4, which reveals that RBP4 promotes VSMC proliferation partly associated with the JAK2 pathway. The scratch wound-healing assay showed that gaps were nearly closed in RBP4-overexpressing VSMCs, whereas gaps remained large in control wells and wells exposed to siRNA-RBP4, AG490, and 1,25(OH)_2_D (50 nM, 48 h). Furthermore, gaps were visible in wells exposed to 1,25(OH)_2_D (25 nM, 24 h) (25 nM,48 h) (50 nM, 24 h), respectively (Figures 6(a) and 6(c)). In flow cytometric analysis, RBP4 overexpression was found to stimulate an increase in cells in the S phase, which implied that RBP4 could potentially induce migration in VSMCs. Furthermore, supplementation with vitamin D resulted in an increased percentage of cells in the G0/G1 phase in a dose-dependent manner (Figure 6(d)). The results of flow cytometric analysis further revealed that VSMCs transfected with overexpressed RBP4 had a significantly lower apoptosis rate compared to cells treated with 50 nM 1,25(OH)_2_D (Figure 6(e)). These findings suggest that vitamin D reduced the abnormal proliferation and migration of VSMCs caused by RBP4 under high glucose condition.

### 3.5. RBP4 Upgrades the Expressions of JAK2, STAT3, cylinD1, and Bcl-2, Which Can be Inhibited by Vitamin D Intervention

**Figure 7:**
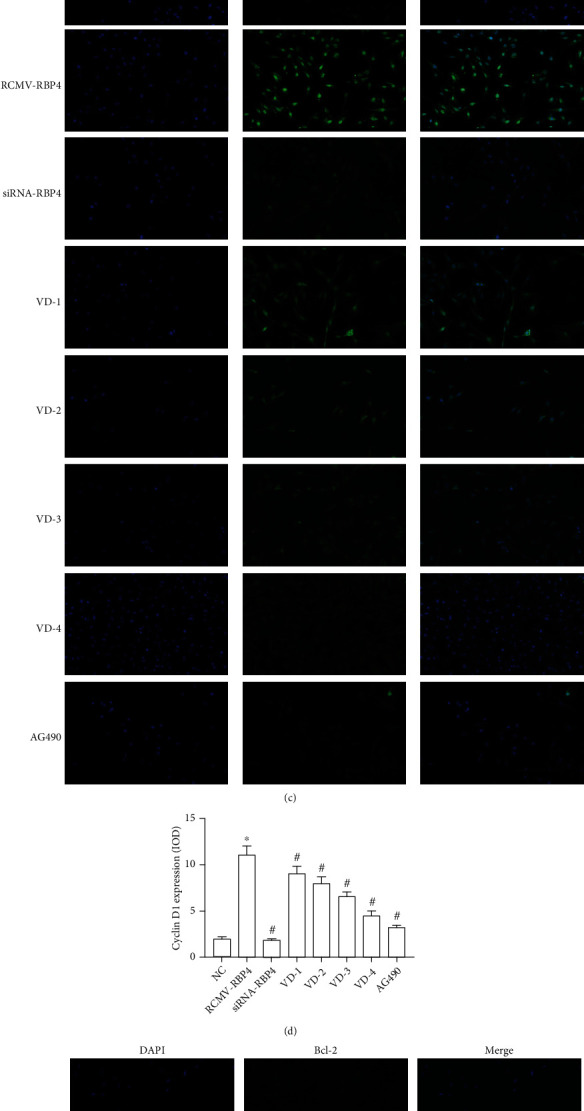
RBP4 can promote VSMCs proliferation and migration under high glucose condition, which can be inhibited by vitamin D. (a) The expressions of JAK2, STAT3, cyclinD1, and Bcl-2 were analyzed by western blot (^∗^*P* < 0.05 for the RCMV-RBP4 group compared to the NC group, ^#^*P* < 0.05 for siRNA-RBP4-, AG490-, and vitamin D-treated groups compared to RCMV-RBP4). (b) The expressions of JAK2, STAT3, cyclinD1, and Bcl-2 were analyzed by qRT-PCR (^∗^*P* < 0.05 for the RCMV-RBP4 group compared to the NC group, ^#^*P* < 0.05 for siRNA-RBP4-, AG490-, and vitamin D-treated groups compared to RCMV-RBP4). (c) Immunofluorescence was performed to detect cyclinD1 (green) in the nucleus, and blue indicates DAPI-stained nuclei (scale bar, 25 *μ*m). (d) The corresponding OD values of cyclinD1are presented (^∗^*P* < 0.05 for the RCMV-RBP4 group compared to the NC group, ^#^*P* < 0.05 for siRNA-RBP4-, AG490-, and vitamin D-treated groups compared to RCMV-RBP4). (e) Immunofluorescence was performed to detect Bcl-2 (green) in the cytoplasm, and blue indicates DAPI-stained nuclei (scale bar, 25 *μ*m). (f) The corresponding OD values of Bcl-2 are presented (^∗^*P* < 0.05, for the RCMV-RBP4 group compared to the NC group, ^#^*P* < 0.05 for siRNA-RBP4-, AG490-, and vitamin D-treated groups compared to RCMV-RBP4).

To evaluate the signaling pathway associated with RBP4 and vitamin D, several critical molecules were examined in the present study. Western blot showed that the phosphorylation levels of JAK2 and STAT3 were increased dramatically in VSMCs incubated with RCMV-RBP4 compared with those in VSMCs treated with siRNA-RBP4 (Figure 7(a)). Furthermore, a similar elevation of JAK2 and STAT3 mRNA levels were also observed in the VSMCs (Figure 7(b)). cyclinD1 and Bcl-2 mRNA levels as well as the protein expressions were increased significantly in group RCMV-RBP4 compared with group NC and group siRNA-RBP4. Moreover, vitamin D was reported to lower the expressions of JAK2, STAT3, Bcl-2, and cyclinD1 in a dose-dependent manner in both western blot and qRT-PCR analysis (Figures 7(a) and 7(b)). Cell immunofluorescence assays indicated that fluorescence intensities of JAK2, STAT3, cyclinD1, and Bcl-2 were brighter in RBP4-overexpressing cells compared to cells treated with siRNA-RBP4 or vitamin D (Figures 7(c)–7(f)).

## 4. Discussion

Diabetic macroangiopathy is identified as a major cause of death in diabetic patients and significantly reduces their quality of life. One of the major contributors to diabetic macroangiopathy is the alterations in vascular homeostasis due to the dysfunction of vascular smooth muscle cell [26]. JAK2 is a nonreceptor tyrosine kinase which phosphorylates and activates STAT3, leading to its dimerization and translocation to the nucleus where STAT3 binds to downstream targets such as cyclinD1 and promotes the proliferation of VSMCs. Thus, the JAK2/STAT3 signaling pathway seems to be one of the critical pathways regulating VSMC proliferation and resulting in the development of metabolic diseases [27, 28]. In this study, the expressions of JAK2, STAT3, cyclinD1, and Bcl-2 in the serum as well as in aortic tissue were significantly elevated in diabetic atherosclerotic rats. To further validate the effect of the JAK2 pathway on the migration and proliferation of VSMCs, AG490, a JAK2-specific inhibitor, was used in the present study. The results indicated that the proliferation abilities of VSMCs were limited compared to control cells, which was in accordance with previous viewpoints.

RBP4, the specific vitamin A carrier in the circulation, is secreted primarily from the liver and adipose tissues. As a newly identified adipokine, RBP4 was suggested to be associated with the pathogenesis of insulin resistance and T2DM. Transgenic overexpression of human RBP4 in rat was found to induce insulin resistance [4]. Conversely, heterozygous and homozygous RBP4 knockout rats exhibited improved insulin sensitivity [29]. Several studies have suggested a potential association between elevated RBP4 levels and the development of diabetes complicated with cardiovascular diseases [30, 31]. However, the literatures have not reached a consensus on this issue [32]. In our study, atherosclerosis of the aorta was observed in HE staining after injection of recombinant RBP4 in diabetic rats, which suggests that RBP4 may be involved in the occurrence of diabetic macroangiopathy. The role of RBP4 in the diabetic atherosclerotic process is connected to an increased level of proinflammatory cell surface adhesion molecules, an unfavourable proatherogenic plasma lipoprotein profile, and insulin resistance [33–35]. Both our previous studies and the present study revealed that RBP4 is associated with IR, inflammation, and glucolipid metabolism, which is in agreement with the results of most previous research [36, 37]. However, it should be pointed out that only a few studies have been conducted on the potential role of RBP4 in the proliferation and migration of VSMCs. Previous research revealed that RBP4 could potentially promote the proliferation of VSMCs [38]. However, they failed to explore and illustrate the concrete mechanisms of RBP4 involved in the proliferation of VSMCs. Adipocytes generate endocrine hormones which engage the JAK/STAT signaling pathway in target tissues [39], implying that JAK2/STAT3 is an adipocyte-derived signaling pathway. Trovati et al. reported that leptin can affect VSMC proliferation and vascular endothelial cell function through the JAK/STAT pathway [40]. In addition to leptin, other JAK inducing factors including IL-6 and prolactin are also produced by adipocytes [41]. Li et al. [42] found that RBP4 can significantly promote the hyperinsulinism-induced proliferation of VSMCs via JAK2/STAT3. Randolph and Ross showed that the treatment of RBP4 for cultured adipocytes triggers the activation of JAK2 and STAT, phosphorylation of stimulated by retinoic acid gene 6 (STRA6), and the subsequent upregulation of suppressor of cytokine signaling 3 (SOCS3), leading to the inhibition of insulin responses [43]. Similarly, the injection of recombinant RBP4 in mice led to the phosphorylation of JAK2 and STAT and upregulation of SOCS3 expression in adipose and muscle tissues [44]. Our previous study had confirmed that RBP4 is associated with the JAK2/STAT3 signaling pathway [23], which may contribute to the development of diabetic macroangiopathy. In the present study, the levels of p-JAK2, p-STAT3, cyclinD1, and Bcl-2 increased after the rats were intraperitoneally injected with recombinant RBP4. Moreover, they were all positively associated with RBP4, indicating that RBP4 participates in the formation of atherosclerosis by the JAK2/STAT3 signaling pathway. To explore if the RBP4/JAK2/STAT3 pathway could initiate the transformation of VSMCs, cells were incubated with glucose conditions in the presence or absence of RBP4. At the cellular level, we found that RBP4 overexpression significantly increased the proliferation and migration of VSMCs and enhanced the expressions of JAK2, STAT3, cylinD1, and Bcl-2. However, RBP4 knockdown induced the opposite effects, which indicates that elevated RBP4 may contribute to the pathogenesis of diabetic atherosclerosis by the JAK2/STAT3 pathway. Thus, the RBP4/JAK2/STAT3 cascade appears to be a promising novel therapeutic target for diabetic macroangiopathy.

Vitamin D insufficiency may contribute to the pathogenesis of diabetic macroangiopathy by numerous potential pathophysiologic mechanisms. The impacts of vitamin D deficiency are known to cause endothelial dysfunction, dyslipidemia, inflammation, and insulin resistance [45]. Vitamin D supplementation can potentially modulate the oxidative-inflammatory reaction, balance arterial remodelling, and increase insulin sensitivity [46]. In our previous clinical trials, after 12 weeks of vitamin D therapy, the incidence rate of lower extremity arterial disease in the T2DM group and the level of RBP4 both decreased significantly [36]. In the present study, vitamin D supplementation alleviated the progression of aortic diseases in diabetic rats with macroangiopathy and attenuated the ability of cell proliferation and migration in the cellular experiment, suggesting that vitamin D supplementation plays a cardioprotective role. Previous researches have shown that vitamin D can influence the production and function of certain adipokines such as leptin and adiponectin [47, 48]. Nevertheless, only a few studies have explored the relationship between vitamin D and RBP4. In our study, the levels of RBP4 were reduced in diabetic atherosclerotic rats after vitamin D supplementation. Additionally, in line with the findings of our previous studies, RBP4 was negatively correlated with 25(OH)D. Vitamin A receptor and 1,25(OH)_2_D receptor belong to the steroid and thyroid hormone receptor family, and their functions often interfere with each other [49]. Kong et al. found that 1,25(OH)_2_D can inhibit the production of transcription factors and the accumulation of lipid during adipocyte differentiation [50]. Metheniti et al. [51] showed that the expression of 25(OH)D was decreased in ultraobese young females and was negatively correlated with RBP4. We therefore speculated that vitamin D might play a pivotal role in the progression of diabetic atherosclerosis partly by influencing the expression of RBP4. Zhang et al. [52] suggested that 1,25(OH)_2_D suppresses the inflammatory responses by inhibiting Th1 cell differentiation and cytokine generation via the JAK/STAT pathway. In this study, we were able to gain insight into the possible mechanisms of the vitamin D and JAK2 pathway. As expected, we found that JAK2 and STAT3 levels of rats in the diabetic atherosclerosis group were negatively correlated with 25(OH)D concentrations and significantly decreased after vitamin D intervention. Similarly, vitamin D lowered the expressions of JAK2, STAT3, cyclinD1, and Bcl-2 in a dose-dependent manner in vitro experiment, indicating that vitamin D supplementation exerts potent inhibitory effects on the proliferation and migration of VSMCs via the RBP4/JAK2/STAT3 pathway.

## 5. Conclusion

Our results cast a new light on how RBP4 induces proliferation and migration of VSMCs via the JAK2/STAT3 signaling pathway and promotes the pathogenesis of diabetic atherosclerosis. We also found that vitamin D supplementation can downgrade RBP4 expression and have a protective role in the development of diabetic macroangiopathy, which is partially associated with suppressing proliferation of VSMCs via the JAK2/STAT3 signaling pathway. Finding new and effective therapeutic targets by thoroughly exploring the regulation and mechanisms of diabetic macrovascular disease is crucial for achieving optimal clinical outcomes. Hence, our present findings provide novel insights into the potential role of RBP4 and vitamin D in the development of diabetic macroangiopathy.

## Figures and Tables

**Table 1:** Primer sequences of target genes and internal reference genes.

Gene	Amplicon size (bp)	Forward primer (5′ →3′)	Reverse primer (5′ →3′)
STAT3	115	GCAATACCATTGACCTGCCG	AACGTGAGCGACTCAAACTG
RBP4	131	GCGAGGAAACGATGACCACT	TGGGGTCACGAGAAAACACA
JAK2	179	ACAAGCAGGACGGGAAGGTC	AATTGGGCCGTGACAGTTGC
Bcl2	102	GAGTACCTGAACCGGCATCT	GAAATCAAACAGAGGTCGCA
*β*-Actin	150	CCCATCTATGAGGGTTACGC	TTTAATGTCACGCACGATTTC
cyclinD1	138	TCAAGTGTGACCCGGACTG	GACCAGCTTCTTCCTCCACTT

STAT3: signal transducer and activator of transcription 3; RBP4: retinol binding protein 4; JAK2: Janus kinase 2; Bcl-2: B cell lymphoma-2.

**Table 2:** Biochemical parameters of rats in different groups.

	NC (*n* = 20)	DM (*n* = 20)	DAS (*n* = 25)	VDAS-1 (*n* = 21)	VDAS-2 (*n* = 20)	VDAS-3 (*n* = 21)	*F*	*P*
Weight (g)	421.30 ± 33.58	553.81 ± 38.06^a^	582.35 ± 37.19^a,b^	554.26 ± 37.92^a,c^	524.86 ± 33.76^a,b,c,d^	518.72 ± 43.68^a,b,c,d^	46.973	≤0.001
MT (*μ*m)	73.15 ± 8.70	92.16 ± 7.05^a^	116.30 ± 9.62^a,b^	111.68 ± 11.03^a,b^	108.19 ± 9.59^a,b,c,^	100.20 ± 9.93^a,b,c,d,e^	59.350	≤0.001
LD (mm)	1.80 ± 0.17	1.76 ± 0.14	2.09 ± 0.18^a,b^	2.09 ± 0.20^a,b^	1.92 ± 0.21^a,b,c,d^	1.63 ± 0.16^a,b,c,d,e^	24.540	≤0.001
LDL-c	0.35 ± 0.03	0.51 ± 0.05^a^	0.57 ± 0.06^a,b^	0.54 ± 0.03^a,b^	0.55 ± 0.05^a,b^	0.48 ± 0.05^a,b,c,d,e^	66.648	≤0.001
TG	0.65 ± 0.04	1.35 ± 0.13^a^	1.82 ± 0.19^a,b^	1.58 ± 0.15^a,b,c^	1.79 ± 0.16^a,b,d^	1.52 ± 0.15^a,b,c,e^	177.841	≤0.001
TC	2.02 ± 0.17	2.24 ± 0.19^a^	2.95 ± 0.19^a,b^	2.37 ± 0.18^a,b,c^	2.44 ± 0.18^a,b,c^	2.21 ± 0.21^a,c,d,e^	66.424	≤0.001
HDL-c	1.06 ± 0.10	1.02 ± 0.06^a^	0.72 ± 0.05^a,b^	0.79 ± 0.07^a,b,c^	0.76 ± 0.06^a,b^	0.94 ± 0.08^a,b,c,d,e^	83.240	≤0.001
HbA1C	5.06 ± 0.36	9.94 ± 1.04^a^	10.80 ± 1.23^a,b^	11.27 ± 0.72^a,b^	9.99 ± 0.97^a,c,d^	9.66 ± 0.85^a,c,d^	121.350	≤0.001
FPG	5.44 ± 0.55	13.39 ± 1.32^a^	13.80 ± 1.51^a^	14.22 ± 1.12^a^	12.49 ± 1.20^a,b,c,d^	12.60 ± 0.97^a,b,c,d^	160.800	≤0.001
FINS	9.73 ± 1.15	14.41 ± 1.39^a^	19.43 ± 1.81^a,b^	19.24 ± 1.77^a,b^	18.85 ± 1.75^a,b^	15.70 ± 1.53^a,b,c,d,e^	117.934	≤0.001
RBP4	15.39 ± 1.24	21.24 ± 1.23^a^	31.33 ± 3.15^a,b^	28.92 ± 2.97^a,b,c^	27.98 ± 3.12^a,b,c^	21.57 ± 1.96^a,c,d,e^	127.386	≤0.001
HOMA-IR	2.35 ± 0.32	8.56 ± 1.05^a^	11.92 ± 1.72^a,b^	12.15 ± 1.36^a,b^	10.44 ± 1.16^a,b,c,d^	8.78 ± 1.05^a,c,d,e^	182.703	≤0.001
25(OH)D	156.77 ± 11.46	124.80 ± 8.56^a^	95.10 ± 8.48^a,b^	448.61 ± 52.19^a,b,c^	544.00 ± 50.22^a,b,c,d^	721.61 ± 89.11^a,b,c,d,e^	656.205	≤0.001
ISI	−3.96 ± 0.15	-5.25 ± 0.13^a^	−5.58 ± 0.15^a,b^	−5.60 ± 0.11^a,b^	−5.45 ± 0.12^a,b,c,d^	−5.28 ± 0.12^a,c,d,e^	475.785	≤0.001

^a^ *P* < 0.05 vs. the NC group, ^b^*P* < 0.05 vs. the DM group, ^c^*P* < 0.05 vs. the DAS group, ^d^*P* < 0.05 vs. the VDAS-1 group, and ^e^*P* < 0.05 vs. VDAS-2 group. NC: control group; DM: diabetes without macrovascular disease group; DAS: diabetic atherosclerosis group; VDAS-1: Diabetic atherosclerosis group treated with vitamin D 0.075 *μ*g kg^−1^ d^−1^; VDAS-2: diabetic atherosclerosis group treated with vitamin D 0.15 *μ*g kg^−1^*μ*d^−1^; VDAS-3: diabetic atherosclerosis group treated with vitamin D 0.3 *μ*g kg^−1^ d^−1^; MT: the media thickness of the artery; LD: lumen diameter; LDL-c: low-density lipoprotein cholesterol; TG: triglycerides; TC: total cholesterol; HDL-c: high-density lipoprotein cholesterol; HbA1C: hemoglobin A1c; FPG: fasting plasma glucose; FINS: fasting insulin; RBP4: retinol binding protein 4; HOMA-IR: homeostasis model assessment of insulin resistance; ISI: insulin sensitivity index.

**Table 3:** Correlation among RBP4, 25(OH)D, and the other indicators in T2DM groups (*n* = 107).

	RBP4		25(OH)D	
	*r*	*P*	*r*	*P*
LDL-c	0.401	≤0.001	-0.294	0.002
TG	0.456	≤0.001	-0.051	0.604
TC	0.616	≤0.001	-0.480	≤0.001
HDL-c	-0.733	≤0.001	0.435	≤0.001
HbA1C	0.162	0.078	-0.201	0.037
FPG	0.177	0.069	-0.273	0.004
FINS	0.655	≤0.001	-0.063	0.522
RBP4	—	—	-0.274	0.004
HOMA-IR	0.606	≤0.001	-0.200	0.039
25(OH)D	-0.274	0.004	—	—
ISI	-0.626	≤0.001	0.345	≤0.001
Weight	0.241	0.013	-0.471	≤0.001
MT	0.587	≤0.001	-0.052	0.592
LD	0.592	≤0.001	-0.348	≤0.001
JAK2	0.753	≤0.001	-0.374	≤0.001
STAT3	0.758	≤0.001	-0.238	0.014
cyclinD1	0.699	≤0.001	-0.365	≤0.001
Bcl-2	0.744	≤0.001	-0.220	0.023

LDL-c: low-density lipoprotein cholesterol; TG: triglycerides; TC: total cholesterol; HDL-c: high-density lipoprotein cholesterol; HbA1C: hemoglobin A1c; FPG: fasting plasma glucose; FINS: fasting insulin; RBP4: retinol binding protein 4; HOMA-IR: homeostasis model assessment of insulin resistance; ISI: insulin sensitivity index; MT: the media thickness of the artery; LD: lumen diameter; JAK2: Janus kinase 2; STAT3:signal transducer and activator of transcription 3; Bcl-2: B cell lymphoma-2.

## Data Availability

The datasets used and/or analyzed during the current study are available from the corresponding author on reasonable request.

## Supplementary Materials

Supplementary Materials Figure S1: RBP4 can promote the proliferation and migration of VSMCs via regulating the JAK2/STAT3 signaling pathway. This mechanism of RBP4 can be inhibited by vitamin D supplementation.
