# Supplementary figures and images for: The Effects of RBP4 and Vitamin D on the Proliferation and Migration of Vascular Smooth Muscle Cells via the JAK2/STAT3 Signaling Pathway
Source: Oxid Med Cell Longev. 2022 Jan 17;2022:3046777. doi: 10.1155/2022/3046777 (PMC8786468; doi:10.1155/2022/3046777)

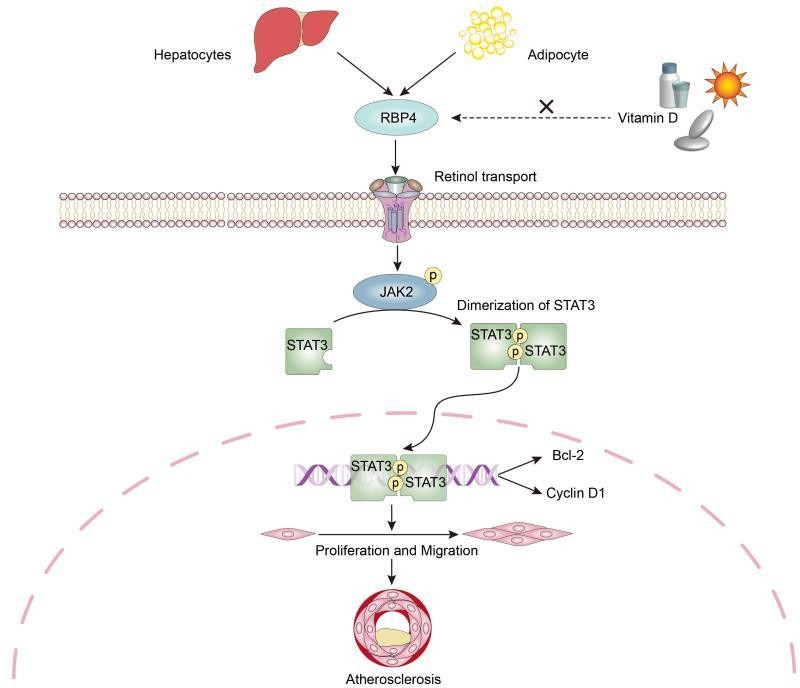

Supplement: Supplementary Materials — Figure S1: RBP4 can promote the proliferation and migration of VSMCs via regulating the JAK2/STAT3 signaling pathway. This mechanism of RBP4 can be inhibited by vitamin D supplementation. [file 3046777.f1.docx]
